# MOF/Polymer Mixed-Matrix Membranes Preparation: Effect of Main Synthesis Parameters on CO_2_/CH_4_ Separation Performance

**DOI:** 10.3390/membranes12040425

**Published:** 2022-04-14

**Authors:** Harun Kulak, Raymond Thür, Ivo F. J. Vankelecom

**Affiliations:** Membrane Technology Group (MTG), Centre for Membrane Separations, Adsorption, Catalysis and Spectroscopy for Sustainable Solutions (cMACS), Faculty of Bioscience Engineering, KU Leuven, Celestijnenlaan 200F, P.O. Box 2454, 3001 Leuven, Belgium; harun.kulak@kuleuven.be (H.K.); raymond.thur@kuleuven.be (R.T.)

**Keywords:** mixed-matrix membranes, CO_2_/CH_4_ separation, synthesis parameter optimization, metal-organic frameworks, polyimide

## Abstract

Design and preparation of mixed-matrix membranes (MMMs) with minimum defects and high performance for desired gas separations is still challenging as it depends on a variety of MMM synthesis parameters. In this study, 6FDA-DAM:DABA based MMMs using MOF-808 as filler were prepared to examine the impact of multiple variables on the preparation process of MMMs, including variation in polymer concentration, filler loading, volume of solution cast per membrane area, solvent type used and solvent evaporation rate, and to identify their impact on the CO_2_/CH_4_ separation performance of these membranes. Solvent evaporation rate proved to be the most critical synthesis parameter, directly influencing the performance and visual appearance of the membranes. Although less dominantly influencing the MMM performance, polymer concentration and solution volume also had an important role via control over the casting solution viscosity, particle agglomeration, and particle settling rate. Among all solvents studied, MMMs prepared with chloroform led to the best performance for this polymer-filler system. Chloroform-based MMMs containing 10 and 30 wt.% MOF-808 showed 73% and 62% increase in CO_2_ permeability, respectively, without a decrease in separation factor compared to unfilled membranes. The results indicate that enhanced gas separation performance of MMMs strongly depends on the cumulative effect of various synthesis parameters rather than individual impact, thus requiring a system-specific design and optimization.

## 1. Introduction

Mixed-matrix membranes (MMMs) are hybrid membrane materials consisting of (nano)particles embedded in a polymeric matrix [1,2]. Owing to their hybrid nature, MMMs are theorized to benefit from the advantages from both porous fillers (efficient separation of gas molecules) and polymers (cheap and good processability). At the same time, they circumvent the brittleness and high costs associated with inorganic membranes and the mediocre gas separation performance of unfilled polymeric membranes [3]. Even though MMMs are currently still waiting for their commercial breakthrough, they have been widely studied in the past decades, leading to truly exceptional performances [4,5,6,7,8,9,10]. Especially for CO_2_/CH_4_ separation, as applied in natural gas and biogas upgrading, MMMs have made up a significant portion of the membrane research field [11,12,13]. The bulk of the published MMM research has focused on (i) the improvement of membrane separation performance in terms of gains in separation factor and/or gas permeability [14], (ii) improving the particle–polymer interfacial adhesion [15], and (iii) identification and development of novel materials that can act as efficient filler [16].

Although their hybrid nature enables MMMs to sidestep traditional challenges in polymeric and inorganic membrane development, they encounter new challenges with regard to the particle–polymer interfacial compatibility, the creation of thin supported layers, and absence of clear structure–performance relationships [17,18,19]. Successful development of MMMs with relevant separation performances thus greatly depends on optimizing and controlling the subtle interplay that exists between polymer and particle properties. However, from a practical perspective, an often underexposed but critical aspect in ensuring good MMM performance remains the methodical optimization of MMM preparation parameters for each individual MMM system. Traditional MMM synthesis at lab-scale often involves (i) the preparation of a polymer solution, (ii) addition of the inorganic particles, followed by (iii) solution casting after which solvent evaporation under (inert) atmosphere will result in the formation of a solid MMM film [20]. During this process, the chosen synthesis parameters influence a wide range of physicochemical properties, including the polymer–particle affinity, particle agglomeration and colloidal stability, polymer solution viscosity, and particle settling rate [21,22].

In this work, five key synthesis parameters (i.e., polymer concentration, casting solution volume, nanoparticle concentration, solvent evaporation rate, and solvent type) are chosen for investigation as they all impact one or more of the above mentioned physicochemical properties. These parameters are systematically investigated with respect to their impact on the MMM performance (i.e., CO_2_/CH_4_ separation factor and CO_2_ permeability) in an attempt to identify the most relevant parameters in the MMM synthesis process. 6FDA-DAM:DABA (6FDD) was selected as polymeric matrix as it is a polyimide with excellent chemical and thermal stability and solvent compatibility [23]. Moreover, it is a widely used benchmark polymer in research [23,24,25,26]. MOF-808 nanoparticles were chosen as filler material, as this is a versatile and stable MOF that provides a platform for introducing different types of functionalization in the MMM [27]. MOF-808 exhibits a large surface area, high pore volume, and versatile physical and chemical properties [28]. Owing to these excellent features, it has been widely studied for various adsorption, catalysis, and membrane-based separation applications [29,30,31,32,33,34]. However, a very limited number of studies focuses on its use in gas separation processes [27,35,36]. In addition to such advantages of MOF-808, another determining factor in the selection of the filler material was the fact that it does not have preferential adsorption sites for the gases studied. Here, it was crucial to consider the trade-off between membrane permeability and selectivity in order to prevent any misinterpretation in data analysis as we aim to understand the impact of MMM synthesis parameters on the gas separation performance. If a highly CO_2_-selective MOF was incorporated, the nonselective voids and defects that might possibly be formed at the polymer-MOF interphase would be compensated by the intrinsic selectivity of the filler. Hence, it was aimed at reducing the influence of the incorporated filler on the separation performance to a negligible level. In this way, the actual effect of MOF incorporation can be examined better through the evaluation of permeability values while the separation factor is expected to remain constant.

## 2. Materials and Methods

### 2.1. Chemicals

6FDA-DAM:DABA (9:1, 6FDD) co-polyimide was kindly provided by Fujifilm Manufacturing Europe B.V. (Tilburg, The Netherlands). Tetrahydrofuran (THF), dichloromethane (DCM), chloroform, 2-butanone (methylethylketone (MEK)), and formic acid (FA) were supplied by Acros (Geel, Belgium). Zirconyl chloride octahydrate (ZrOCl_2_·8H_2_O) was purchased from abcr GmbH (Karlsruhe, Germany) and 1,3,5-benzenetricarboxylate (BTC) from J&K Chemicals (Lommel, Belgium). CO_2_ (>99.999%) and CH_4_ (>99.999%) gases were purchased from Air Liquide (Herenthout, Belgium) and used as delivered.

### 2.2. MOF Synthesis

MOF-808 was synthesized as described in our previous study [37]. First, 182 mL H_2_O was used to dissolve 5.08 g (24.2 mmol) BTC and 23.4 g (72.8 mmol) ZrOCl_2_·8H_2_O in a 500 mL round-bottom flask and subsequently 26.8 mL (712 mmol) FA was added. After 15 min of stirring, the mixture was heated to 100 °C under reflux for 5 h in an oil bath. The resulting MOF sludge was washed four times with demi water and three times with ethanol. In between washing steps, the samples were centrifuged (4000 rpm, 10 min) and the supernatant was decanted. Finally, the washed MOF was dried in a vacuum oven at 70 °C overnight.

### 2.3. Membrane Synthesis

6FDD co-polyimide was dissolved in a respective solvent (THF, chloroform, DCM, or MEK) by stirring overnight to produce 3, 4, 5, 7, 10, or 15 wt.% polymer solutions. These solutions were prepared in two different volumes (small and large) to attain membranes with different thicknesses and those cast from 4 g of polymer solutions were denoted as S-version while the ones cast from 6 g of polymer solutions were denoted as L-version. The solutions were then poured into Teflon Petri dishes (diameter 6 cm) in a N_2_-filled glove bag. In order to examine the effect of evaporation rate (fast, medium, and slow, respectively), three different approaches were applied: (i) the solvent was allowed to evaporate without any preventive cover, (ii) solvent evaporation was slowed down by placing a funnel upside-down (with volume of 200 mL, bottom diameter of 10 cm, and top diameter of 1.5 cm) over the dishes, and (iii) evaporation was further slowed down by covering this funnel outlet with alumina foil.

For the preparation of MMMs, a prescribed amount of MOF-808 was dispersed in the respective solvent in an ultrasonic bath for 30 min and then the polymer was added in three steps (in equal amounts at 2 h intervals) according to a priming protocol [38]. Amounts of 10 and 30 wt.% MOF-808 loadings were used for a representative comparison of low and high filler loadings, respectively. The MOF loading (wt.%) indicates the mass ratio of MOF-808 to the polymer in the casting solution (Equation (1)). The MOF-808/6FDD MMMs were formed by casting their solutions under the same conditions as the unfilled polymeric membranes. The complete compositions of the membrane casting solutions prepared are provided in Table S1.

(1) $$Filler\;loading\;\left(\mathrm{wt}.\%\right)=\left(\frac{m_{filler}}{m_{filler}+m_{polymer}}\right)\times 100$$

For thermal annealing, the solidified membranes were heated to 100 °C at a rate of 5 °C/min and an isothermal condition was maintained for 1 h. The temperature was then increased till 180 °C using a heating rate of 5 °C/min. Subsequently, it was held for 6 h at this temperature and then cooled overnight to room temperature naturally. Coupons with a diameter of 20 mm were cut from dried membranes and the thickness of resulting membrane coupons was measured using a Mitutoyo IP65 digital micrometer. For each coupon, measurements were performed on five different locations and the average value obtained. Small and large casting solution volumes resulted in final membrane thickness of 33–196 μm and 50–291 μm, respectively, depending on the preparation conditions. In general, L-version membranes were found to be 1.4–1.7 times thicker than S-version counterparts.

### 2.4. Characterization

#### 2.4.1. X-ray Diffraction (XRD)

Crystallinity of the MOF was assessed by XRD. A Malvern PANalytical Empyrean diffractometer was used to measure samples in transmission mode over a 1.3–45° 2θ range. A PIXcel3D solid-state detector was used and X-rays were generated by a Cu anode (Cu Kα_1_: 1.5406 Å; Cu Kα_2_: 1.5444 Å).

#### 2.4.2. Scanning Electron Microscopy (SEM)

Membrane cross-sections and particle morphology and size were examined with a Philips XL30 FEG scanning electron microscope. All samples were coated with a gold/palladium layer.

#### 2.4.3. Attenuated Total Reflectance-Fourier Transform Infrared Spectroscopy (ATR-FTIR)

ATR-FTIR spectroscopy was applied to investigate the functional groups in MOF and membrane samples. A Varian 670 FTIR imaging spectrometer was used with a diamond ATR crystal and single point MCT detector.

#### 2.4.4. Thermogravimetric Analysis (TGA)

TGA was performed with Q500 thermogravimetric analyzer from TA instruments. Samples were heated in N_2_ atmosphere from room temperature to 800 °C at a heating rate of 5 °C/min.

#### 2.4.5. Differential Scanning Calorimetry (DSC)

DSC measurements were performed on the membranes, using a TA instruments DSC Q2000. Polymer thermal history was erased by heating the samples up to 430 °C. Next, the membranes were cooled to 330 °C and reheated to 430 °C.

### 2.5. Gas Separation Performance

The gas separation performance of the membranes was assessed with an in-house developed gas permeation set-up, described in detail elsewhere [39,40]. The CO_2_/CH_4_ separation factors (α*) are determined by a GC analysis of the permeate composition through Equation (2).

(2) $$A_{CO_{2}/CH_{4}}{}^{*}=\frac{y_{CO_{2}}/y_{CH_{4}}}{x_{CO_{2}}/x_{CH_{4}}}$$

where *y*_*CO*_2__and *y*_*CH*_4__ are the mole fractions of CO_2_ and CH_4_ in the permeate, respectively, and *x*_*CO*_2__ and *x*_*CH*_4__ are the mole fractions of CO_2_ and CH_4_ gases in the feed, respectively.

The mixed-gas CO_2_ permeability of the membranes is determined with the constant-volume-varying-pressure method, in which a MKS Baratron sensor measures the pressure change as a function of time (*dp*/*dt*) in a pre-calibrated downstream volume.

(3) $$P_{CO_{2}}=10^{10}\times \frac{y_{CO_{2}}\times V\times L}{x_{CO_{2}}\times p_{up}\times A\times R\times T}\times \frac{dp}{dt}$$

with *P_i_* the gas permeability (Barrer), *y_i_* the mole fraction of the component in the permeate, *x_i_* the mole fraction of the component in the feed, *V* the downstream volume (cm^3^), *A* the membrane permeation area (1.91 cm^2^), *L* the membrane thickness (cm), *T* the operating temperature (K), *p_up_* the upstream pressure (cmHg), *R* the gas constant (0.278 cm^3^ cmHg/cm^3^(STP) K), and *dp*/*dt* the pressure increase (cmHg/s).

## 3. Results and Discussion

To optimize the synthesis method of 6FDD-based MMMs, the following synthesis parameters were systematically investigated to examine their effects on the characteristics and performance of the prepared MMMs: (i) polymer concentration, (ii) filler loading, (iii) volume of the solution cast per membrane area, (iv) solvent evaporation time after casting, and (v) solvent type used. It is important to note that only one type of polymer (6FDD) and filler (MOF-808) was considered here to manage the complexity of the experimental matrix. The different MMMs are denoted as P*x*-*y*-M*z*, with *x* the polymer concentration in the casting solution, *y* the casting solution volume (S, S-version; L, L-version) and *z* the MOF-808 loading. For example, the S-version of an MMM cast from a solution containing 10 wt.% polymer and 30 wt.% MOF-808 is denoted as P10-S-M30.

Before the preparation of the MMMs, the crystal structure and morphology of the synthesized MOF-808 was verified. XRD and SEM (Figure S1) indicated that crystalline and octahedral MOF particles with an average size of 350 nm were obtained in good agreement with the literature [27,41]. N_2_ sorption revealed a type I isotherm with a BET surface area of 2304 m^2^/g and a pore volume of 0.76 cm^3^/g, well in line with previously reported BET values for MOF-808 [42,43].

### 3.1. THF-Based Membranes: Effect of Polymer and MOF Concentration, Casting Solution Volume, and Solvent Evaporation Rate

#### 3.1.1. Visual Assessment

The membranes were first visually assessed based on their brittleness, curling, and homogeneity issues as shown in Table 1. The MMMs cast from 3 wt.% polymer solutions were, in general, brittle or suffered from severe curling and non-homogeneities, regardless of any synthesis parameter. Their brittle and delicate nature resulted in handling difficulties. Membrane curling is a consequence of an asymmetric distribution of tensions over the membrane cross-section [44]. This can have multiple reasons, such as settling of fillers during drying or a skin layer that has rigidified too quickly before all solvent from the bottom parts is released. A 3 wt.% polymer concentration was thus inadequate to make MMMs of 6FDD.

When the polymer concentration was increased to 5 wt.%, two key parameters that predominantly determined the MMMs visual appearance were the evaporation rate of the solvent after casting and the MOF loading in the MMMs. Well-formed membranes were obtained with slower solvent evaporation rates, whereas fast evaporation of the solvent always rendered brittle or severely curled membranes. A medium solvent evaporation rate resulted in well-formed membranes when a low loading of MOF was used but failed to deliver qualitative MMMs for 30 wt.% MOF loadings (with the exception of P5-S-M30). In general, for higher filler loadings, the effects of solvent evaporation rate were found to be more pronounced.

The MMMs cast from 10 wt.% polymer solutions also confirmed that reducing the evaporation rate is imperative to make good MMMs. Especially for high filler content, membranes that could be cut into coupons for subsequent gas separation testing could be obtained only when the evaporation rate was slowed down, as the fast solvent evaporation always rendered brittle or severely curled membranes. Furthermore, the casting solutions which contained 10 wt.% polymer and 30 wt.% MOF-808 led to very brittle and non-homogeneous membranes (with the exception of P10-S-M30 with slow evaporation, further confirming the importance of slow solvent evaporation rate). Accordingly, preparation of L-version membranes with a combination of high MOF loading for 10 wt.% polymer solutions (indicated as NA in Table 1) was not attempted.

Considering the results of MMMs cast from solutions containing 10 wt.% polymer, only low filler loadings were used while preparing membranes from 15 wt.% polymer solutions. Even though the strongly increased viscosity of the polymer casting solution at 15 wt.% polymer impeded the casting procedure, good membranes were produced with low MOF loading and slow evaporation rate, similar to those of membranes cast from solutions containing 10 wt.% polymer. In contrast, attempts to prepare membranes from 20 wt.% polymer solutions failed due to the very high viscosity of the solution. For all polymer concentrations, the thickness of the cast layer did not affect the overall quality of the membrane. However, in the case of the membrane cast from 10 wt.% 6FDD solution containing 30 wt.% MOF loading, substantially better MMMs were obtained when using a small instead of a large casting solution volume.

#### 3.1.2. Gas Separation Performances

**Figure 1:**
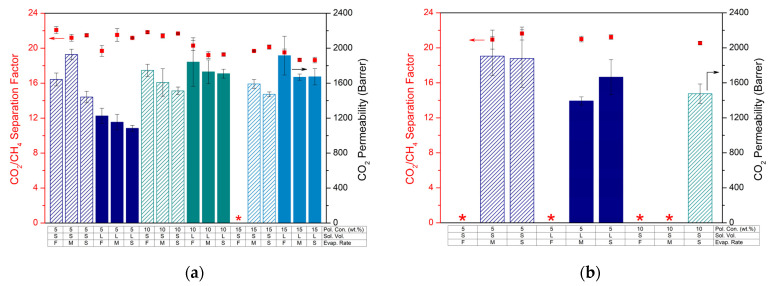
Mixed-gas CO_2_/CH_4_ separation factors (dots) and CO_2_ permeabilities (bars) of MOF-808/6FDD MMMs containing (**a**) 10 wt.% and (**b**) 30 wt.% filler, which were prepared under different synthesis conditions. “S” (“L”) implies the small (large) solution volume. “F” indicates fast evaporation, while “M” and “S” represent medium and slow evaporation rates, respectively. Red stars correspond to the membranes with no valid results due to the brittleness or defects.

After the visual assessment of MMMs prepared with different synthesis parameters in THF, their gas separation performances were tested for an equimolar CO_2_/CH_4_ gas mixture at 35 °C and 2 bar. Figure 1 shows the mixed-gas CO_2_/CH_4_ separation factors and CO_2_ permeabilities for MMMs prepared under various conditions. For 10 wt.% MOF-808 loadings, increasing the polymer concentration above 5 wt.% resulted in MMMs which displayed equal or lowered CO_2_/CH_4_ separation factors (Figure 1a). The separation factors of the membranes cast from solutions containing 15 wt.% polymer were significantly lower than those of 5 and 10 wt.% polymer concentrations. Additionally, no effect of polymer concentration on CO_2_ permeability could be observed. With respect to casting solution volume, 5 wt.% polymer solutions led to higher permeabilities for S-version membranes, while the permeability values did not differ significantly for 10 and 15 wt.% casting solutions when S- and L-versions were compared. On the other hand, the separation factors appeared to be higher in general for S-version membranes. No clear trend could be identified with respect to the effect of THF evaporation rate on the MMM CO_2_/CH_4_ separation factor. However, slow evaporation of THF in general led to a lower CO_2_ permeability compared to fast evaporation. This can possibly be understood by faster evaporation leading to polymer chains that do not have sufficient time to rearrange properly and reach an optimal packing [45]. For MMMs prepared with 30 wt.% MOF loading, Figure 1b clearly represents the importance of the solvent evaporation rate. Regardless of any other parameter studied in this work, it was not possible to obtain defect-free MMMs with high filler content if the solvent is evaporating rapidly. Furthermore, even though CO_2_/CH_4_ separation factors remain almost constant, membranes showing higher gas permeability can be obtained by lowering the polymer concentration and casting solution volume [46,47].

**Figure 2:**
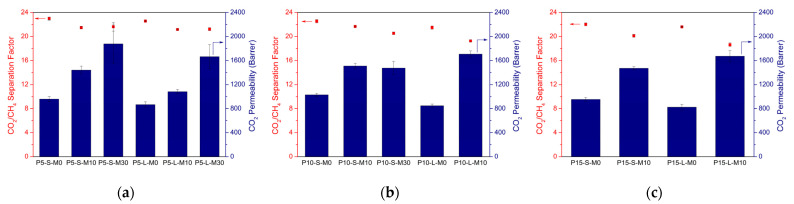
Mixed-gas CO_2_/CH_4_ separation factors (dots) and CO_2_ permeabilities (bars) of unfilled 6FDD membranes and corresponding MMMs cast from (**a**) 5 wt.%, (**b**) 10 wt.%, or (**c**) 15 wt.% polymer solutions with different combinations of synthesis parameters. “PX” represents the polymer concentration of the solutions while “S” and “L” indicate the small and large solution volume, respectively. “MX” corresponds to the amount of MOF loading (wt.%).

For a better comparison of the performance improvement of the membranes upon filler incorporation, similar gas separation measurements were performed for unfilled 6FDD membranes prepared with different polymer concentrations and casting solution volumes at slow evaporation rate. Figure 2 represents the results of these measurements together with their MMM counterparts, containing different filler loadings and cast from small or large casting volumes.

In general, slight decreases can be observed in the separation factors of the membranes upon MOF incorporation, while permeabilities increased significantly. The change in casting solution polymer concentration had no significant impact on the performance of the unfilled polymeric membranes, whereas the casting solution volume influenced their performance adversely with increasing volume. For the performance of MMMs, however, low polymer concentration and small solution volume led to more favorable results for CO_2_ separation, consistent with the results in Figure 1.

### 3.2. Effect of Solvent Type

Another very important parameter during MMM synthesis is the solvent type used, as it directly influences the viscosity of the solution, the filler sedimentation rate, dispersion and stabilization of the MOF, and the time required for the complete evaporation of the solvent and thus solidification of the membrane. For this purpose, and based on the findings in the previous section, four different volatile solvents (DCM, chloroform, MEK, and THF) that can readily dissolve 6FDD and have different boiling points, chemical interactions with the MOF surface, and densities were considered. MMMs from casting solutions containing 5 wt.% polymer concentration with different MOF loadings and solution volumes were prepared using these solvents under slow evaporation rate. Some of the important physical properties of the solvents studied are listed in Table 2.

#### 3.2.1. Visual Assessment

Membranes were first assessed based on their visual appearances, as shown in Table 3. The MOF particles were dispersed well in DCM-based solutions and the membranes prepared with DCM showed a good visual quality at first glance. As DCM is the solvent with the lowest boiling point, it was expected to avoid particle sedimentation during solvent evaporation as the viscosity of casting solution rises rapidly after casting, thus slowing down sedimentation [46]. The DCM-cast membranes containing a high amount of MOF were too brittle to process into coupons. Probably, the polymer chains did not experience sufficient time to rearrange, pack properly, and seal well around the particles due to the high volatility of DCM, leaving voids behind. In addition, DCM possibly did not allow good dispersion for this high amount of filler. The use of chloroform ensured a better dispersion of MOF-808 particles during stirring without creating an overly viscous solution, owing to its relatively high density. Moreover, it allowed the membranes to dry in an optimal time span, and produced homogeneous and flexible membranes, even at 30 wt.% MOF loading. When MEK was used as the solvent, however, all membranes displayed severe curling, even the unfilled polymeric membranes, as it requires a much longer time for complete solvent evaporation after casting due to its higher boiling point. Apart from curling, MEK also resulted in a poor MOF dispersion, probably due to its low density and viscosity. The Stokes–Einstein theory correlates the sedimentation rate of particles with viscosity of fluid and density difference between particle and surrounding fluid (Equation (4)) [50]. Accordingly, sedimentation increases if the viscosity is low and the density difference between particle and solution is high, as with MEK, whereas it can be impeded with increasing viscosity and decreasing density difference, as with chloroform.

(4) $$v=\frac{2\;r_{p}^{2}\;\left|\rho_{p}-\rho_{f}\right|\;g}{9\;\mu_{f}}$$

where *v* is the sedimentation rate of particles, *r_p_* the radius of the particles, *ρ_p_* the density of the particles, *ρ_f_* the fluid density, *g* the gravitational acceleration, and *μ_f_* the fluid viscosity.

#### 3.2.2. Gas Separation Performances

**Figure 3:**
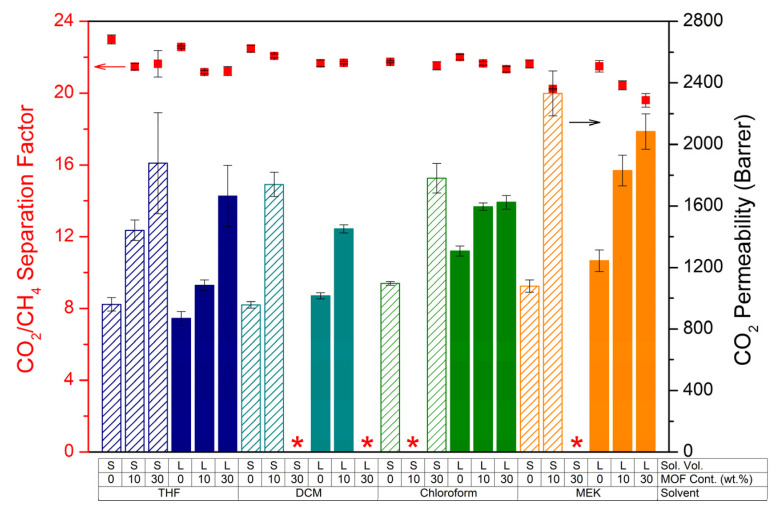
CO_2_/CH_4_ separation factors (**dots**) and CO_2_ permeabilities (**bars**) of 6FDD and MMMs cast from 5 wt.% polymer solutions prepared with different solvents. “S” and “L” indicate the small and large solution volume, respectively. Red stars correspond to the membranes with no valid results due to the brittleness or defects.

Figure 3 compares the mixed-gas CO_2_/CH_4_ separation factors and CO_2_ permeabilities of the membranes prepared from THF, DCM, chloroform, and MEK. As described above, THF-based membranes (both S- and L-version) showed a slightly lower separation factor by 7 and 6%, respectively, after introduction of MOF-808 for both casting solution volumes upon addition of 10 and 30 wt.% MOF compared to their unfilled counterparts, most probably due to the formation of nonselective interfacial voids between filler particles and polymer matrix. Thanks to the introduction of the MOF pores, and possibly also because some defects were created, a significant increment in permeability was observed for these MMMs.

At 10 wt.% MOF loading, good results were obtained for DCM-based membranes. MMMs showed a decrease in CO_2_/CH_4_ separation factor of only −2% compared to the unfilled membrane for the S-version, and no change in separation factor for the L-version. CO_2_ permeability increased with 82% for the S-version membrane while a 43% increase was denoted for the L-version. However, at 30 wt.% loadings, DCM rendered brittle membranes that could not be processed.

Chloroform-based MMMs yielded the best results with almost no decline in separation factor, indicating an optimal incorporation of the MOF in the polymer matrix. Although the preparation of the MMM with 10 wt.% MOF loading failed, the MMM containing 30 wt.% MOF displayed an equal separation factor (~22) to that of the reference membrane (S-version). For the L-version, the MMMs resulted in almost unchanged separation factors upon 10 and 30 wt.% MOF incorporation (2 and 3% lower, respectively). CO_2_ permeabilities of the membranes containing 30 wt.% MOF were improved by 62% and 24% for S- and L-versions, respectively.

Finally, the gas permeation experiments revealed that MMMs prepared with MEK showed the highest improvement in permeability (more than twofold increase compared to the unfilled membrane for the S-version). However, it is apparent that MEK-based membranes suffered from a more pronounced decrease in separation factor as a consequence of possible defect formation discussed above.

#### 3.2.3. Characterization of MMMs

XRD patterns of the membranes prepared from different solvents (THF, DCM, chloroform, and MEK) proved that the solvents used in this study do not lead to structural changes within the framework of MOF-808 and that the crystalline structure of all particles was well preserved after incorporation into the polymer matrix (Figure S2). SEM cross-sections of the MMMs also indicated the successful incorporation of the filler with homogeneous distribution in the polymer matrix for all four types of solvents (Figure S3). However, it is also observable that a minor portion of the incorporated fillers form agglomerates and sediment when THF and MEK were used as a solvent. These defective interphases can be linked to the decline in separation factor of these MMMs. Figure S4 shows the ATR-FTIR spectra of the membranes. The characteristic bands of the co-polyimide are observed at 1790 cm^−1^ and 1722 cm^−1^ for asymmetric and symmetric modes of (C=O) stretching, respectively, and at 1358 cm^−1^ for stretching vibration of (C−N) [24]. On the other hand, all MMMs exhibited characteristic peaks related to MOF-808. Among these peaks, those at 1622, 1570, and 1382 cm^−1^ belong to the vibrational bands in BTC linker while the most noticeable features located at 655 and 452 cm^−1^ are assigned to Zr−*μ*_3_−O and Zr−*μ*_3_−OH stretching vibrations, respectively [38,51]. According to the thermograms presented in Figure S5, the use of different solvents in MMM preparation affected neither the solvent presence in the membrane after synthesis nor the thermal stability of the materials. For all membranes prepared using different solvents, a thermal decomposition temperature (T_d_) varying between 494 and 498 °C was observed (Table S2). In addition to the similar T_d_ values obtained, DSC results presented in Figure S6 indicate that the glass transition temperatures (T_g_) of the membranes were not affected by the solvent type used during the fabrication process of the MMMs. As given in Table S2, the T_g_ values of the 10 wt.% MMMs prepared from all solvents are similar.

### 3.3. Optimized Synthesis of Chloroform-Based Membranes

Based on the promising results using chloroform in Section 3.2, a screening study for chloroform-based MMMs was performed to be able to identify the optimal synthesis parameters for the MMM fabrication.

#### 3.3.1. Visual Assessment

As shown in Table 4, S-version membranes always rendered curling and non-homogeneities for a 10 wt.% MOF-808 loading. For a 30 wt.% filler loading, however, well-formed membranes were obtained with increasing the polymer concentration. When the membranes were prepared in L-version, MMMs containing low filler loading still suffered from curling and non-homogeneity with 3 and 4 wt.% polymer concentrations, whereas 5 and 7 wt.% polymer concentrations produced well-formed membranes. For high MOF loadings, almost every polymer concentration resulted in good MMMs in the L-version.

#### 3.3.2. Gas Separation Performance

**Figure 4:**
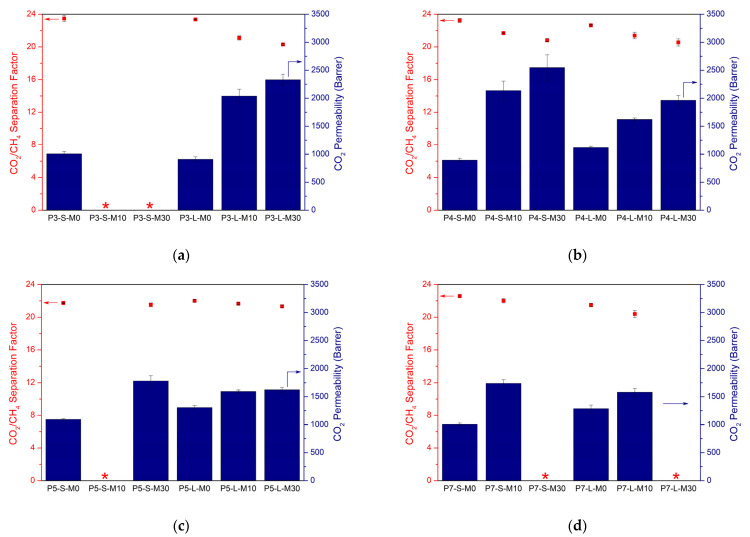
Mixed-gas CO_2_/CH_4_ separation factors (dots) and CO_2_ permeabilities (bars) of chloroform-based unfilled 6FDD membranes and corresponding MMMs cast from (**a**) 3 wt.%, (**b**) 4 wt.%, (**c**) 5 wt.%, and (**d**) 7 wt.% polymer solutions with different combinations of synthesis parameters. “PX” represents the polymer concentration of the solutions while “S” and “L” imply the small and large solution volume, respectively. “MX” indicates the amount of MOF loading (wt.%). Red stars correspond to the membranes with no valid results due to the brittleness or defects.

Figure 4 shows the gas separation performances of these membranes prepared with different polymer concentration, filler loading, and casting solution volume. For the membranes cast from a solution containing 3 wt.% polymer, no defect-free membranes could be obtained in the S-version. Even though MMMs prepared from a 4 wt.% polymer solution overcame this problem, they experienced a drop in separation factors, similar to those of the L-version membranes cast from solutions with 3 or 4 wt.% polymer, again indicating the existence of defects at the polymer–particle interface and non-ideal incorporation of the MOF into the polymer matrix.

The obtained data suggest that the S-version does not outperform the L-version when using chloroform, unlike in the THF-based system. Most probably, the low boiling point of chloroform reduces the time required for complete solvent evaporation. As the solvent evaporation occurs faster at the edges of the Petri dishes than at the center [52], radially outer parts of the membranes detach from the surface while the inner parts still stick to the dish. This outcome creates curling and non-homogeneity of the membrane, ultimately influencing the MMM performance.

Similarly, chloroform causes the performance decrease of the MMMs to begin at lower polymer concentrations as compared with THF. As can be seen in Figure 4d, membranes cast from 7 wt.% polymer solutions suffer from defects upon high MOF incorporation. On the other hand, for MMMs containing 10 wt.% MOF, the L-version led to a 5% decrease in separation factor while no change was observed for the S-version compared to unfilled membrane counterparts. CO_2_ permeabilities of these membranes were improved by 23 and 73% for L- and S-versions, respectively. Overall, the most remarkable enhancement in MMM performance was observed with the S-version of the 7 wt.% polymer solution for 10 wt.% MOF loading, while the S-version of the 5 wt.% polymer solution led to the best performance improvement for 30 wt.% MOF loading. This observed increase in gas permeability is comparable to the 6FDD-based MMMs reported in the literature [53,54,55]. However, those membranes consist of low filler content (≤20 wt.%) and often lack in maintaining the enhanced separation performance upon higher filler loadings due to the defected MMM morphology, whereas a significant improvement was obtained upon 30 wt.% MOF-808 incorporation in this study.

#### 3.3.3. Characterization

XRD patterns illustrated in Figure S2 indicate that characteristic features of MOF-808 were preserved and the crystallinity remained intact during MMM preparation. Further, the characteristic vibrational bands belonging to both 6FDD and MOF-808 were observed in ATR-FTIR spectra of all MMMs (Figure S4), similar to previous findings in Section 3.2.3. TGA traces of the MMMs showed that MOF-808 incorporation does not affect the thermal decomposition of the membranes and the high thermal stability is maintained upon filler addition (Figure S5). The thermal profiles were similar for the membranes prepared with different polymer concentrations, filler loadings, and solution volumes, as anticipated. The major backbone degradation was observed after 490 °C for all membrane samples, with a decreased T_d_ with increasing filler content (Table S2). Additionally, a shoulder was observed in the respective DTG curve of the membrane containing 30 wt.% MOF-808 (Figure S5f) as a consequence of the different decomposition mechanisms of the polymer and filler phases. Based on DSC analysis, T_g_ was elevated by approximately 6 °C for MMMs compared to unfilled 6FDD membrane upon 10 and 30 wt.% MOF incorporation (Table S2), indicating an enhanced polymer rigidification as the incorporated filler particles restrict the polymer chain mobility [56]. This is, however, not visible in the gas permeation data. The increase in T_g_ upon filler incorporation also indicates a good compatibility between MOF-808 and 6FDD [57]. No effect in T_g_ was observed for MMMs with varying filler amount or casting solution volume. Some defects may have been introduced, as indicated by the diminished improvement observed in MMM permeability at high filler loadings (Figure 4). In SEM cross-sections of the membranes (Figures S7 and S8), a more nodular network was observed for MMMs compared with unfilled 6FDD membrane as a result of interfacial stress between polymer matrix and filler phases [38,58].

### 3.4. Overall Comparison

**Figure 5:**
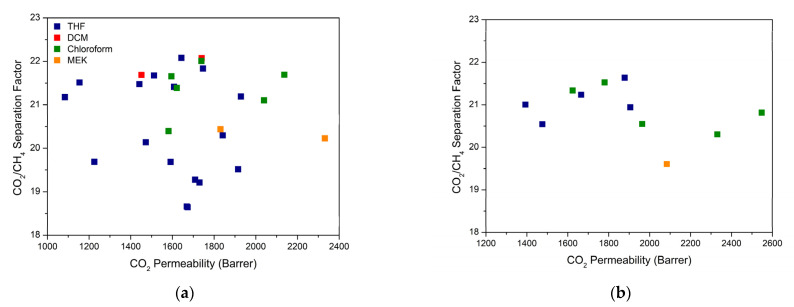
Permeability/separation map showing mixed-gas data measured at 2 bar and 35 °C with 50/50 (vol.%) CO_2_/CH_4_ mixture for MOF-808/6FDD MMMs containing (**a**) 10 wt.% and (**b**) 30 wt.% filler, which were prepared under different synthesis conditions, labeled here by solvent from which the membranes were cast.

A final overall comparison of the gas separation performance of the MOF-808 MMMs is given in Figure 5. For the same MOF/polymer system, entirely different separation performances were obtained for each MMM prepared under different synthesis parameters, proving how the MMM performance can be drastically impacted by simply adjusting the synthesis condition. Clearly, the effect of a governing parameter can be boosted or lessened by another parameter, indicating that an enhanced separation performance strongly relies on a cumulative impact of these parameters rather than on their individual effect. Overall, the data suggest that it is possible to improve the separation performance of MMMs based on the same filler-polymer pair by varying and optimizing the synthesis parameters, which is often overlooked and underestimated in the literature. Such synthesis effects can thus clearly overrule the success of combining the right filler with the right polymer for a given separation process. In general, for the polymer-MOF combination considered in this work, the chloroform-based samples are situated more in the top-right corner of the graph, thus proving its preferred use to prepare this type of MMMs.

## 4. Conclusions

MOF-808/6FDA-DAM:DABA MMMs were selected as a case study to demonstrate the effect of different synthesis parameters on the corresponding MMM performance. Different polymer concentrations, filler loadings, casting solution volumes, solvent types, and solvent evaporation rates were investigated, and the resulting MMMs were tested for CO_2_/CH_4_ gas separation performance.

For THF-based membranes, the rate of solvent evaporation proved to be the most important parameter in MMM synthesis. Better-quality membranes were obtained as the evaporation rate was reduced, as fast evaporation rendered brittle or severely curled membranes. In addition, the MMMs containing 30 wt.% MOF were found to be more prone to adverse effects of fast evaporation compared to the MMMs with lower loading. However, slow evaporation generally resulted in lower CO_2_ permeability, but this could be overcome by reducing the polymer concentration and casting solution volume. With respect to polymer concentration in the casting solution, concentrations above 5 wt.% resulted in MMMs which displayed equal or lowered CO_2_/CH_4_ separation factors, but lower polymer concentration led to higher gas permeabilities. On the other hand, even though the casting solution volume did not influence the overall visual quality, S-version membranes scored higher separation factors than L-version membranes. Volatile solvents rendered brittle membranes as a consequence of too fast solvent evaporation, while the curling issue became prominent for membranes prepared with the less volatile solvents.

MMMs prepared with chloroform resulted in a remarkable performance with almost no decline in separation factor, indicating an optimal incorporation of the MOF particles into the polymer matrix. For high MOF loadings, well-formed membranes were produced for almost each polymer concentration using a large solution volume. However, defect-free membranes could not be obtained with 3 wt.% polymer solutions when a small solution volume was adopted. Even though MMMs cast from 4 wt.% polymer solutions overcome this problem, they experienced reduced separation factors. Nevertheless, when prepared at optimal polymer concentration of 5 wt.%, they did not show any decrease in separation factor but higher CO_2_ permeability upon 30 wt.% MOF-808 incorporation. These results suggest that the influence of various synthesis parameters should be taken into consideration for the design and development of novel MMMs with improved performance, which can prevail over the selection of promising filler-polymer pairs for MMM applications.

## Figures and Tables

**Table 1:** Visual assessment of THF-based MMMs prepared with different combinations of synthesis parameters ^1^.

		S-Version						L-Version					
		10 wt.% MOF Loading			30 wt.% MOF Loading			10 wt.% MOF Loading			30 wt.% MOF Loading		
	Evap. Rate	Fast	Medium	Slow	Fast	Medium	Slow	Fast	Medium	Slow	Fast	Medium	Slow
Pol. Con.													
3 wt.%		−	−	+	−	+	+	−	+	+	−	−	−
5 wt.%		+	++	++	−	++	++	+	++	++	−	++	++
10 wt.%		+	++	++	−	−	++	+	++	++	NA	NA	NA
15 wt.%		−	++	++	NA	NA	NA	+	++	++	NA	NA	NA

^1^ (−) brittle membrane, (+) membrane with curling and/or non-homogeneity issues, (++) well-formed membrane, and (NA) not attempted.

**Table 2:** Physical properties of solvents used in this study [48,49].

Solvent	Boiling Point (°C)	Density ^a^ (g/cm^3^)	Viscosity ^b^ (mPa·s)	Vapor Pressure ^c^ (kPa)	Surface Tension ^d^ (mN/m)	Relative Polarity	Dispersion of Filler ^e^
THF	65	0.8892	0.456	21.6	26.40	0.207	Fair
DCM	40	1.3266	0.413	58.2	27.20	0.309	Good
Chloroform	61	1.4832	0.537	26.2	26.67	0.259	Good
MEK	80	0.8054	0.405	12.6	23.97	0.327	Poor

^a^ Density at a temperature of 20 °C. ^b^ Dynamic viscosity at 25 °C. ^c^ Vapor pressure at 25 °C. ^d^ Surface tension at 25 °C. ^e^ Visual rating of MOF-808 dispersion quality while stirring the casting solutions.

**Table 3:** Visual assessment of MMMs prepared with different solvents ^1^.

	S-Version			L-Version		
Solvent	0 wt.% MOF	10 wt.% MOF	30 wt.% MOF	0 wt.% MOF	10 wt.% MOF	30 wt.% MOF
THF	++	++	++	++	++	++
DCM	++	++	−	++	++	−
Chloroform	++	+	++	++	++	++
MEK	+	+	+	+	+	+

^1^ (−) brittle membrane, (+) membrane with curling and/or non-homogeneity issues, and (++) well-formed membrane.

**Table 4:** Visual assessment of chloroform-based MMMs prepared with different combinations of synthesis parameters ^1^.

Polymer Concentration	S-Version			L-Version		
	0 wt.% MOF	10 wt.% MOF	30 wt.% MOF	0 wt.% MOF	10 wt.% MOF	30 wt.% MOF
3 wt.%	++	+	+	++	+	++
4 wt.%	++	+	+	++	+	++
5 wt.%	++	+	++	++	++	++
7 wt.%	++	+	++	+	++	+

^1^ (+) membrane with curling and/or non-homogeneity issues, and (++) well-formed membrane.

## Data Availability

Not applicable.

## Supplementary Materials

The following are available online at https://www.mdpi.com/article/10.3390/membranes12040425/s1, Table S1: Composition of casting solutions used for membrane preparation. Table S2: Thermal decomposition (T_d_) and glass transition (T_g_) temperatures of 6FDD-based MMMs. Figure S1: (a) XRD pattern, (b) SEM image, (c) N_2_ adsorption–desorption isotherm, and (d) the distribution of incremental pore volume of MOF-808. Figure S2: XRD patterns of 6FDA-DAM:DABA MMMs prepared with different synthesis parameters. “PX” represents the polymer concentration of the casting solutions while “L” (“S”) implies the large (small) solution volume. “MX” corresponds to the amount of MOF loading (wt.%) and solvent name indicates the solvent used during the preparation of that membrane. Figure S3: SEM cross-sections of 6FDA-DAM:DABA MMMs prepared with different solvents. All membranes are cast from large volume of solution with 5 wt.% polymer concentration and 10 wt.% MOF-808 loading. Figure S4: FTIR spectra of 6FDA-DAM:DABA MMMs prepared with different synthesis parameters: (a) effect of solvent type, (b) effect of polymer concentration and casting solution volume, and (c) effect of filler amount. “PX” represents the polymer concentration of the casting solutions while “L” (“S”) indicates the large (small) solution volume. “MX” corresponds to the amount of MOF loading (wt.%). Figure S5: TGA (a,c,e) and DTG (b,d,f) curves of 6FDA-DAM:DABA MMMs prepared with different synthesis parameters: (a,b) effect of solvent type, (c,d) effect of polymer concentration and casting solution volume, and (e,f) effect of filler amount. “PX” represents the polymer concentration of the casting solutions while “L” (“S”) indicates the large (small) solution volume. “MX” corresponds to the amount of MOF loading (wt.%). Figure S6: DSC curves of 6FDA-DAM:DABA MMMs prepared with different synthesis parameters: (a) effect of solvent type, (b) effect of filler amount and casting solution volume. “PX” represents the polymer concentration of the casting solutions while “L” (“S”) indicates the large (small) solution volume. “MX” corresponds to the amount of MOF loading (wt.%). Figure S7: SEM cross-sections of 6FDA-DAM:DABA MMMs prepared with different polymer concentration and casting solution volume. All membranes are cast from solution with 10 wt.% MOF-808 loading. Figure S8: SEM cross-sections of 6FDA-DAM:DABA MMMs prepared with different filler loadings. All membranes are cast from large volume of solution with 5 wt.% polymer concentration.
