# Supplementary material for: MOF/Polymer Mixed-Matrix Membranes Preparation: Effect of Main Synthesis Parameters on CO2/CH4 Separation Performance
Source: Membranes (Basel). 2022 Apr 14;12(4):425. doi: 10.3390/membranes12040425 (PMC9026548; doi:10.3390/membranes12040425)
Supplement: Supplementary file 1 [file membranes-12-00425-s001.zip › membranes-1688088-supplementary.pdf]

Supporting Information for:

MOF/Polymer Mixed-Matrix Membranes Preparation: Effect of Main Synthesis Parameters on CO_2/CH_4 Separation Performance

Harun Kulak, Raymond Thür and Ivo F. J. Vankelecom *

Membrane Technology Group (MTG), Centre for Membrane Separations, Adsorption, Catalysis and
Spectroscopy for Sustainable Solutions (cMACS), Faculty of Bioscience Engineering, KU Leuven, Celestijnenlaan 200F, P.O. Box 2454, 3001 Leuven, Belgium;
harun.kulak@kuleuven.be (H.K.);
raymond.thur@kuleuven.be (R.T.)
* Correspondence: ivo.vankelecom@kuleuven.be

Table S1. Composition of casting solutions used for membrane preparation.†

Sample	Polymer (g)	Solvent (g)	Filler (g)
P3-S-M0	0.12	3.88	-
P3-S-M10	0.12	3.88	0.014
P3-S-M30	0.12	3.88	0.052
P3-S-M0	0.18	5.82	-
P3-S-M10	0.18	5.82	0.020
P3-S-M30	0.18	5.82	0.078
P4-S-M0	0.16	3.84	-
P4-S-M10	0.16	3.84	0.018
P4-S-M30	0.16	3.84	0.068
P4-L-M0	0.24	5.76	-
P4-L-M10	0.24	5.76	0.027
P4-L-M30	0.24	5.76	0.103
P5-S-M0	0.20	3.80	-
P5-S-M10	0.20	3.80	0.023
P5-S-M30	0.20	3.80	0.086
P5-L-M0	0.30	5.70	-
P5-L-M10	0.30	5.70	0.034
P5-L-M30	0.30	5.70	0.128
P7-S-M0	0.28	3.72	-
P7-S-M10	0.28	3.72	0.032
P7-S-M30	0.28	3.72	0.120
P7-L-M0	0.42	5.58	-
P7-L-M10	0.42	5.58	0.047
P7-L-M30	0.42	5.58	0.180
P10-S-M0	0.40	3.60	-
P10-S-M10	0.40	3.60	0.044
P10-S-M30	0.40	3.60	0.172
P10-L-M0	0.60	5.40	-
P10-L-M10	0.60	5.40	0.067
P10-L-M30	0.60	5.40	0.257
P15-S-M0	0.60	3.40	-
P15-S-M10	0.60	3.40	0.067

P15-S-M30	0.60	3.40	0.257
P15-L-M0	0.90	5.10	-
P15-L-M10	0.90	5.10	0.100
P15-L-M30	0.90	5.10	0.386

† "PX" represents the polymer concentration of the solutions while "S" ("L") indicates the small (large) solution volume. "MX" corresponds to the amount of MOF loading (wt.%).

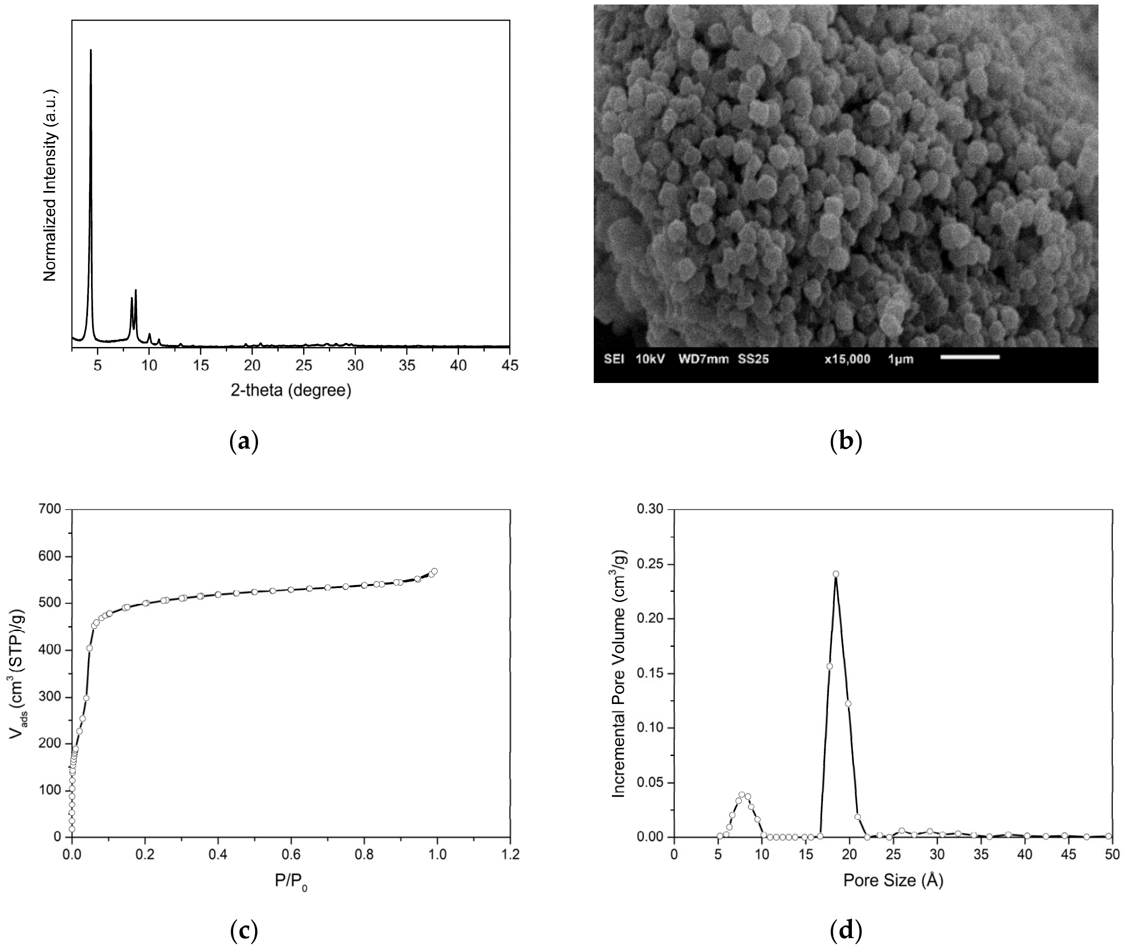

Figure S1. (**a**) XRD pattern, (**b**) SEM image, (**c**) N_2 adsorption-desorption isotherm, and (**d**) the distribution of incremental pore volume of MOF-808.

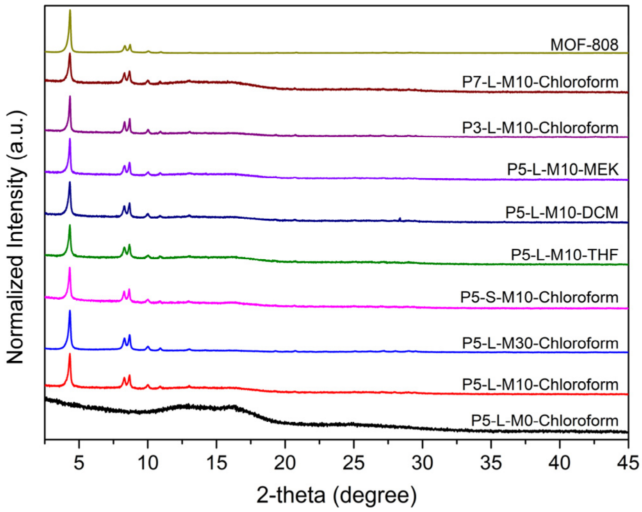

Figure S2. XRD patterns of 6FDA-DAM:DABA MMMs prepared with different synthesis parameters. "PX" represents the polymer concentration of the casting solutions while "L" ("S") implies the large (small) solution volume. "MX" corresponds to the amount of MOF loading (wt.%) and solvent name indicates the solvent used during the preparation of that membrane.

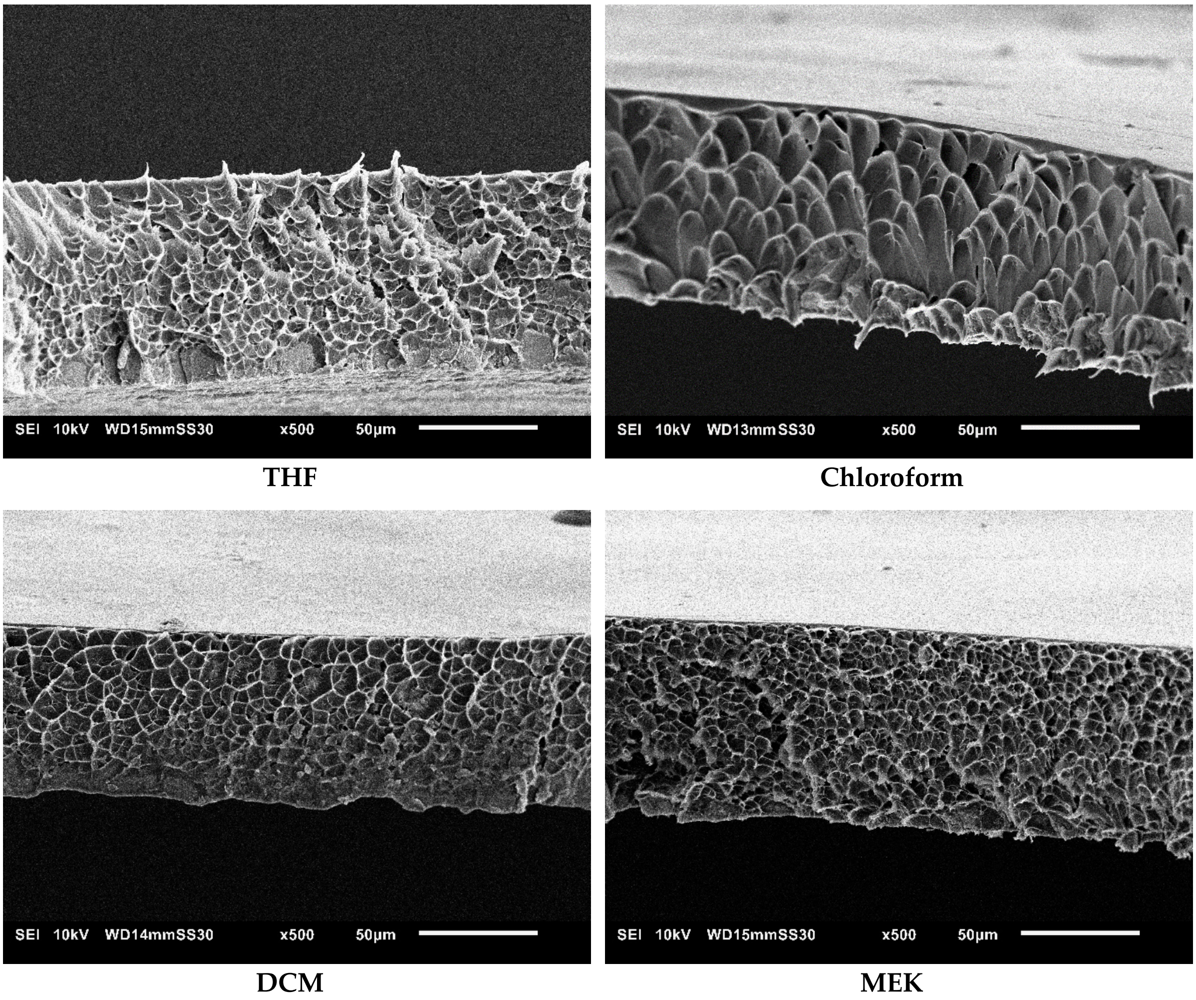

Figure S3. SEM cross-sections of 6FDA-DAM:DABA MMMs prepared with different solvents. All membranes are cast from large volume of solution with 5 wt.% polymer concentration and 10 wt.% MOF-808 loading.

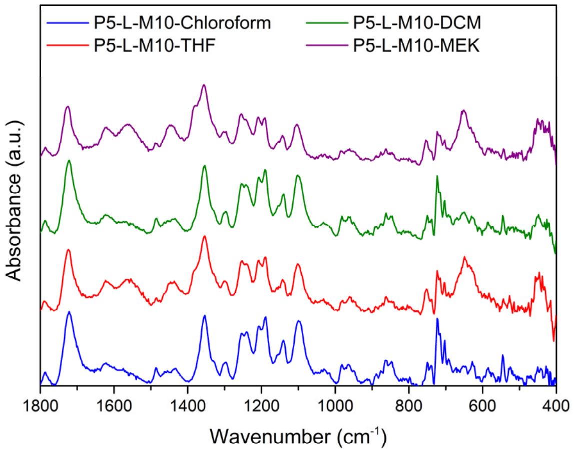

P5-L-M10-Chloroform
P5-L-M10-DCM
P5-L-M10-THF
P5-L-M10-MEK
Absorbance (a.u.)
1800 1600 1400 1200 1000 800 600 400
Wavenumber (cm-1)

(**a**)

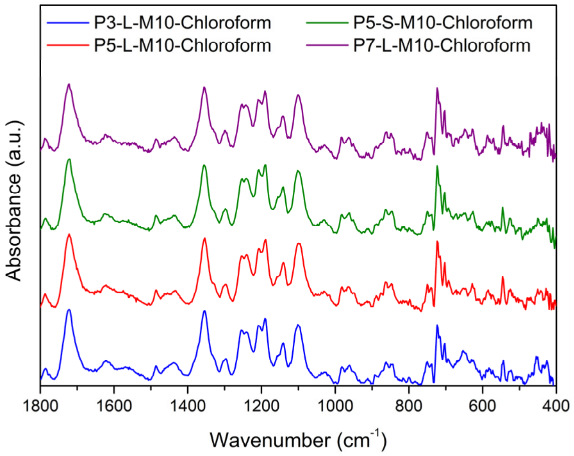

P3-L-M10-Chloroform
P5-S-M10-Chloroform
P5-L-M10-Chloroform
P7-L-M10-Chloroform
Absorbance (a.u.)
1800 1600 1400 1200 1000 800 600 400
Wavenumber (cm-1)

(**b**)

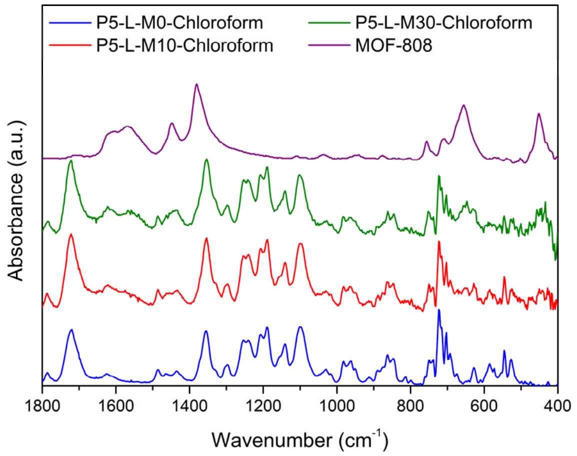

(**c**)

Figure S4. FTIR spectra of 6FDA-DAM:DABA MMMs prepared with different synthesis parameters: (**a**) effect of solvent type, (**b**) effect of polymer concentration and casting solution volume, (**c**) effect of filler amount. “PX” represents the polymer concentration of the casting solutions while “L” (“S”) indicates the large (small) solution volume. “MX” corresponds to the amount of MOF loading (wt.%).

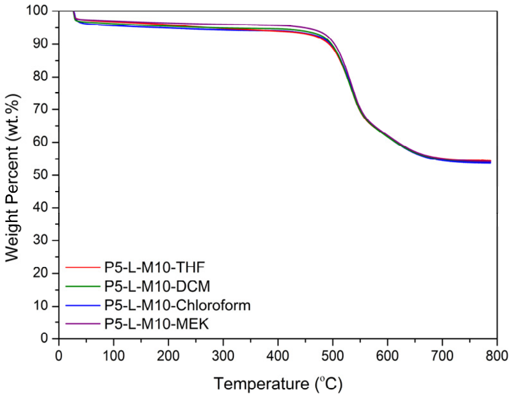

(**a**)

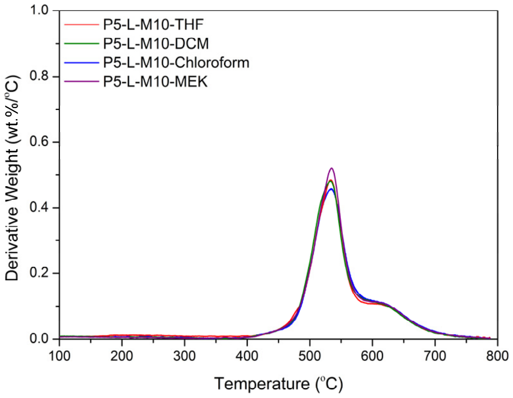

(**b**)

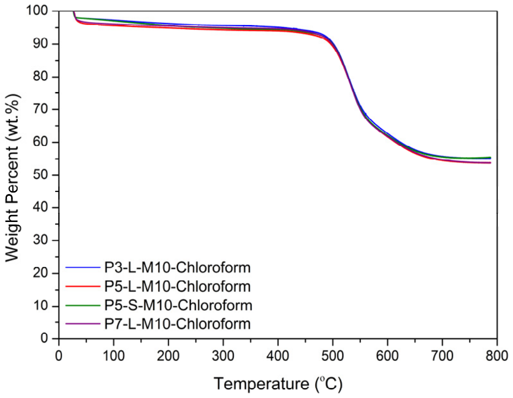

(**c**)

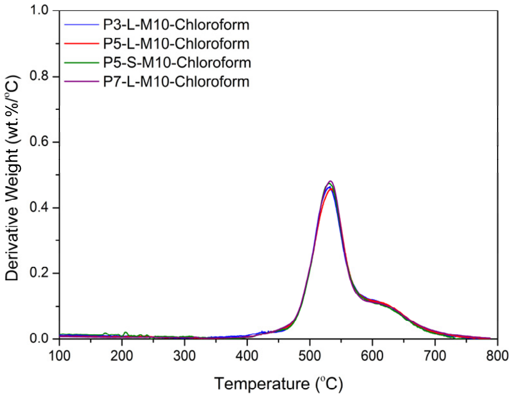

(**d**)

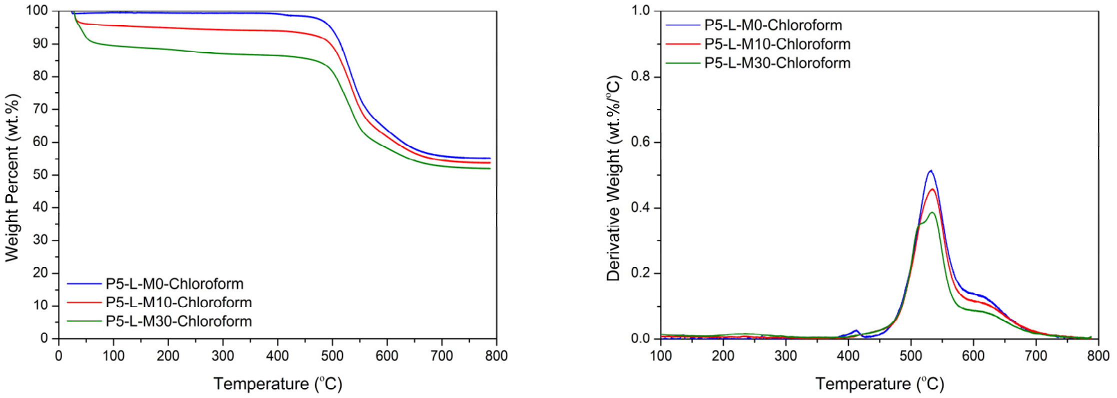

(**e**) (**f**)

Figure S5. TGA (**a**,**c**,**e**) and DTG (**b**,**d**,**f**) curves of 6FDA-DAM:DABA MMMs prepared with different synthesis parameters: (**a**,**b**) effect of solvent type, (**c**,**d**) effect of polymer concentration and casting solution volume, (**e**, **f**) effect of filler amount. "PX" represents the polymer concentration of the casting solutions while "L" ("S") indicates the large (small) solution volume. "MX" corresponds to the amount of MOF loading (wt.%).

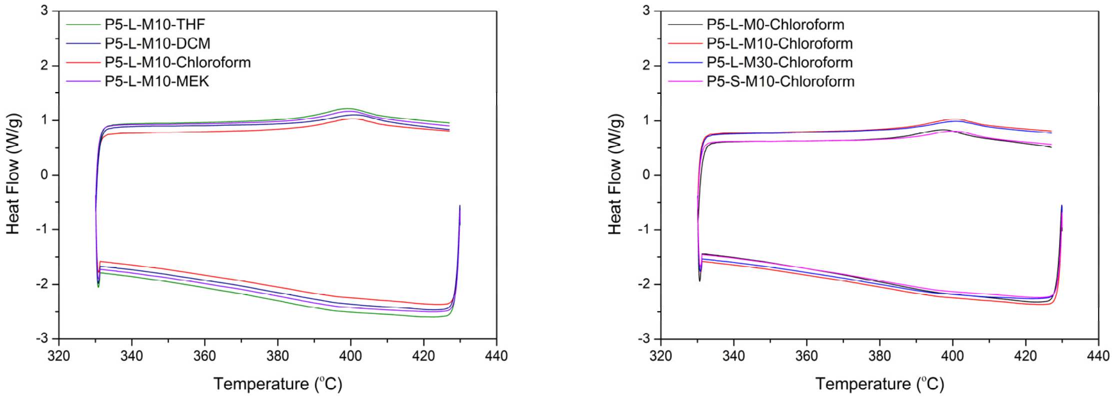

(**a**) (**b**)

Figure S6. DSC curves of 6FDA-DAM:DABA MMMs prepared with different synthesis parameters: (**a**) effect of solvent type, (**b**) effect of filler amount and casting solution volume. "PX" represents the polymer concentration of the casting solutions while "L" ("S") indicates the large (small) solution volume. "MX" corresponds to the amount of MOF loading (wt.%).

Table S2. Thermal decomposition (T_d) and glass transition (T_g) temperatures of 6FDD-based MMMs.

**Sample**	**T_d (°C)**	**T_g (°C)**
P5-L-M0-Chloroform	498	389
P5-L-M10-Chloroform	497	396

P5-L-M30-Chloroform	492	395
P5-S-M10-Chloroform	497	396
P5-L-M10-THF	498	395
P5-L-M10-DCM	494	395
P5-L-M10-MEK	495	396
P3-L-M10-Chloroform	496	NA
P7-L-M10-Chloroform	498	NA

NA: Not Available.

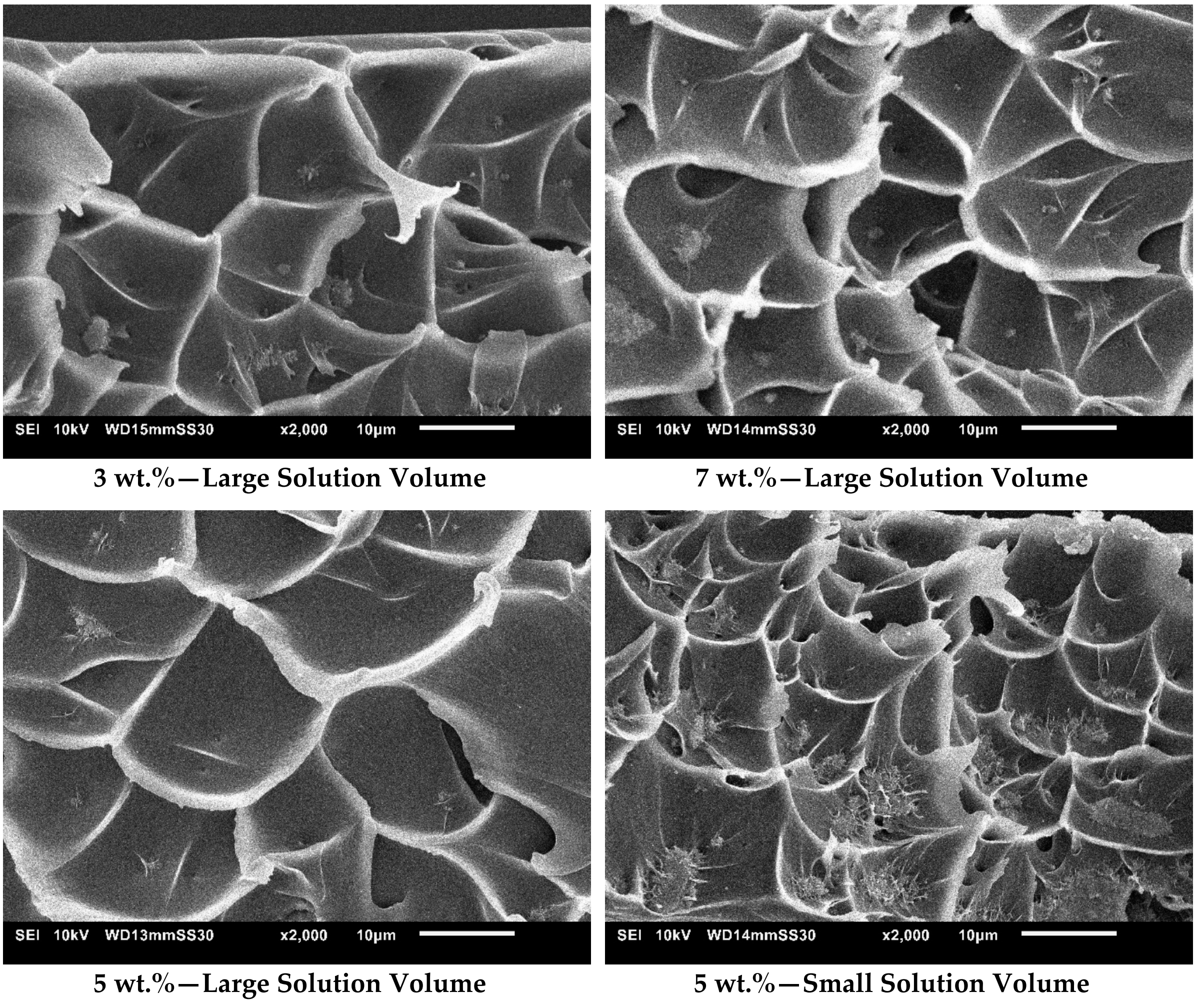

Figure S7. SEM cross-sections of 6FDA-DAM:DABA MMMs prepared with different polymer concentration and casting solution volume. All membranes are cast from solution with 10 wt.% MOF-808 loading.

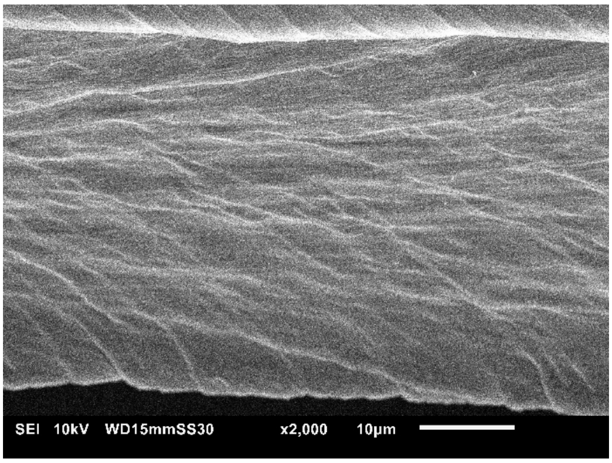

0 wt.% MOF-808

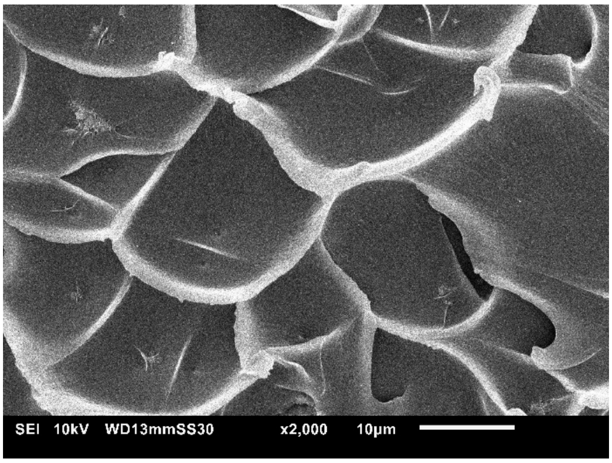

10 wt.% MOF-808

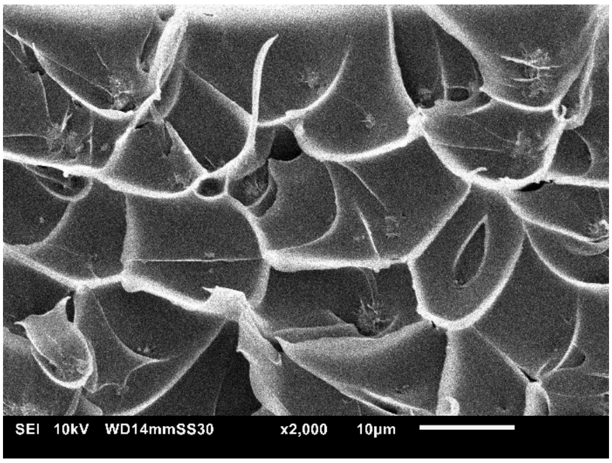

30 wt.% MOF-808

Figure S8. SEM cross-sections of 6FDA-DAM:DABA MMMs prepared with different MOF loadings. All membranes are cast from large volume of solution with 5 wt.% polymer concentration.
